# Parturient Apoplexy: A Discussion of “Milk Fever”1Read before the Nebraska State Veterinary Association.

**Published:** 1900-11

**Authors:** W. A. Thomas

**Affiliations:** Lincoln, Neb.


### PARTURIENT APOPLEXY: A DISCUSSION OF "MILK FEVER."[1]

By W. A. Thomas, D.V.M.,
LINCOLN, NEB.

This disease, or lesion, affecting our domestic animals occurs principally in the cow. It has baffled the skill of veterinarians ever since good cows were known, and not infrequently has the owner of one of these most useful animals had his highest hopes suddenly turned to disappointment and grief. He looked with pride on a noble cow, a fine calf, and thought of the possibilities of an abundance of rich milk. He goes out again too look at his prize, and what has happened? She is prostrated, and already half unconscious, pays no attention to his approach, nor to the calf that plays about the yard.

There is no time to lose. The doctor is called, and everything possible is done to save the animal, but she gradually sinks and dies. The owner wonders if that doctor "knew anything." That is just where we are standing to-day: wondering if the doctors know anything about parturient apoplexy.

Veterinary literature since the commencement of the century teems with descriptions and discussions relative to the disease, and the most eminent veterinary pathologists still appear to be far from unanimous as to its nature. This statement, made by Fleming a quarter of a century ago, still holds good.

In this vaunted day of science new theories are advanced and new treatments invented to relieve the concomitant conditions of this as yet disputed lesion.

Dr. Schmidt, of Denmark, inaugurated the idea that the trouble originated in the udder, and his treatment is to inject a solution of iodide of potassium into the udder through the teats. His paper giving theory, details, treatment, etc., was translated by Dr. Williams and published in the *American Veterinary Review*, covering a space of forty pages. The treatment was very soon taken up by veterinarians throughout the United States. Good practitioners have had varied success with the Schmidt treatment, saving, according to the figures they have given, from 25 to 75 per

[1] Read before the Nebraska State Veterinary Association.

cent. Dr. Schwartzkopf, who is most enthusiastic for the Schmidt treatment, collected data which he presented in a paper before the American Veterinary Association in New York last September, himself losing two cases out of eight, and a third by complications.

The intravenous treatment is comparatively a new one. So far as we can learn, the success with this mode is about equal to the Schmidt.

Instrument-makers have not been slow in aiding the would-be-up-to-date practitioner, as they are already making and advertising instruments for the new treatment.

If the Schmidt theory is correct some one should invent an antitoxin for the purpose of treating the liable subjects before parturition, and produce immunity from such attack.

At the request of this Association I collected data on this subject. The following list of questions were sent to the members of the profession in this State : 1. Time of taking ill after delivery? 2. What was the treatment? 3. Recovery or death? 4. In case of death, give post-mortem, if any. 5. What is your theory as to the pathology?

I received eleven replies. The replies to question No. 1 varied from time during delivery up to ten days after. The replies to the remaining questions were as follows :

1. Used Schmidt's treatment, also hypodermatic injection of strychnine ; tapped the rumen ; magnesia sulphate as cathartic. Two cases—one that received strychnine recovered. Theory on pathology, nervous intoxication.

2. Intravenous injection, hot-packs, catheterization, iod. pot.; with the intravenous treatment have saved 70 per cent. ; none before. Pathology, am inclined to Schmidt's theory.

3. Iod. pot. per mouth, laxatives and stimulants ; no success with Schmidt's mode. Recovery, 50 per cent. Pathology, inclined to believe that the trouble is in what we call the parturition centre in the lumbosacral region, of such nature that the nerves posterior to this cease to act.

4. Intravenous injection ; iod. pot. per mouth ; stimulants ; hot fomentations to loins. Recovery, 75 per cent. Pathology, am undecided.

5. Intravenous injection, hot-packs to body and cold to the head ; eserine hypodermatically, and later strychnine. Of late using the iod. pot. treatment (Schmidt's) in connection with above, and less of the hot and cold applications. Gives no percentage of success and no theory on pathology.

6. Laxative and stimulants, nux vomica and brandy; counter irritation to spine. Died after twenty-eight hours' treatment. Pathology, no theory expressed.

7. Life-root and pulsatilla every two hours. Recovery about 70 per cent. Pathology, no satisfactory theory.

8. Blood-root and pulsatilla every two hours, followed with magnesium sulphate and aromatic stimulants. Had seven cases one very wet week last June; most of them delivered in pasture and all died. Had ten other cases that made good recovery. Pathology, no theory expressed.

9. Sulphate magnesia in one gallon of water, ice on head, stimulating liniment to spine. After acute stage nerve tonics and stimulants. Five out of eight recovered. Schmidt's treatment in five cases, all making good recovery. Pathology: immediately after calving a superabundance of blood in the system, which has been nourishing the fœtus; is divided in these new channels for the production of milk, and the mammary glands at this time are generally stimulated to the utmost capacity, and therefore this blood, instead of being converted into milk, is retained in the system, causing degeneration of the nerves and centres.

10. All that has been recommended. Best success with Schmidt's treatment. Recovery, probably 60 per cent. Pathology: believe it is due to impression on the brain and spinal cord due to ptomaïn-poisoning or bacteria. Do not believe all the fault is in the udder.

11. Epsom salt, common salt, gamboge; three doses of pulsatilla and life-root, and no more. Recovery, 50 to 75 per cent. Pathology: think that there is a large amount of nutriment in the blood that should go to the calf, or milk that is thrown back upon the system, and the large amount of blood that does not find a natural outlet, and is thrown back on the system and influencing the sympathetic system, and I think is owing to the bad circulation of the brain.

In the eleven reports only two give post-mortem conditions. One states: "Brain soft, bloodless and flabby." This animal must have died after a few days' illness, and the lesions thus found were secondary.

Another states: "I have held many post-mortems and have always found thrombosis and embolism in the pelvic, sacral, and also in the mesenteric bloodvessels. Congestion of the various organs, the result, I think, of vasomotor troubles. In the posterior pelvic and sacral regions the various tissues seem bloodless, soft, and flabby, and in a very dry condition."

I wonder if the doctor really meant what he said—*i. e.*, that he found thrombosis and embolism in the pelvic, sacral, and mesenteric bloodvessels, or were they post-mortem clots? If he found thrombosis and embolism, then such conditions must have been preceded by disease of the heart or bloodvessels. An embolism is the obstruction of a bloodvessel by an embolus; an embolus is a clot formed in one place and transported by the blood to another locality; such emboli are ante-mortem conditions.

To recapitulate: Nine gave medicines per mouth; three the Schmidt treatment; one the intravenous treatment; two the intravenous and Schmidt; two ice or cold applications to head.

Of the number who have given medicines per mouth, not one has stated how they were administered, whether by drench or through a tube. If the cow is in a comatose condition, medicines cannot be safely administered by a drench, as they are liable to pass down the trachea, causing asphyxia or choking. The animal that is repeatedly drenched and recovers had but a slight lesion to overcome.

Franck gives a list of over 700 cases, with 60 per cent. recoveries. This is in Europe, where the percentage of recovery is very much reduced on account of the large number of animals slaughtered and used for food that in the United States would have had opportunity to recover.

We see articles upon this subject headed, "Parturient Paresis." This word paresis should not be used in connection with this lesion. We have only to consult lexicographers for the reason.

*Universal Dictionary of the English Language:* "Paresis patho: Insanity, with general paralysis. The loss of motor power is progressive. Those afflicted rarely live more than from one year to three years." *Webster:* "Paresis: Incomplete paralysis affecting motion but not sensation." *Duane:* "Paresis: Incomplete motor paralysis." *Dunglison:* "Paresis: Diminution of motor power; slight incomplete paralysis, affecting motion but not sensation, but by some regarded synonymous with paralysis."

Apoplexy is a sudden diminution or loss of consciousness, sensation, and voluntary motion, usually caused by pressure on the brain. If this pressure is due to congestion or from other causes when removed the animal recovers and is in no manner paralyzed. Then, what is parturient apoplexy? There are already too many answers to this question.

In a typical case, taken ill six hours after delivery, dying ten hours later, without a struggle, I find on post-mortem meningeal congestion of the brain and spinal cord hemorrhage, a clot extending full width of the fourth ventricle, with œdema or water. Can any one doubt that such conditions are sufficient cause for death?

The work of Friedberger and Fröhner, translated from the German and published in 1895, states that the pathological anatomy presents nothing or almost nothing characteristic.

These animals do not die without a cause. The symptoms are constant. They are that of coma, and coma is a symptom of lesions of the brain. Then we must look in the brain for the lesions. These are the primary lesions.

According to the percentages given, our losses are still too great. I would earnestly ask the profession to make post-mortem examinations on the animals we lose, that we may agree on the cause of death. Until then treatment of whatever mode is to a certain extent empirical.

